# The Impact of ISO Certification Procedures on Patient Safety Culture in Public Hospital Departments

**DOI:** 10.3390/healthcare13060661

**Published:** 2025-03-18

**Authors:** Georgia Kyriakeli, Anastasia Georgiadou, Maria Lithoxopoulou, Zoi Tsimtsiou, Vasilios Kotsis

**Affiliations:** 1 Department of Nursing Service, General Hospital Papageorgiou, 564 03 Thessaloniki, Greece; 2 Human Milk Bank, 2nd Department of Neonatology and Neonatal Intensive Care Unit, Aristotle University of Thessaloniki, General Hospital Papageorgiou, 564 03 Thessaloniki, Greece; natgeorg@hotmail.com; 3 2nd Department of Neonatology and Neonatal Intensive Care Unit, School of Medicine, Aristotle University of Thessaloniki, 541 24 Thessaloniki, Greece; lithoxopoulou@auth.gr; 4 Department of Hygiene, Social-Preventive Medicine and Medical Statistics, School of Medicine, Aristotle University of Thessaloniki, 541 24 Thessaloniki, Greece; ztsimtsiou@auth.gr; 5 3rd Department of Internal Medicine, School of Medicine, Aristotle University of Thessaloniki, 541 24 Thessaloniki, Greece; vkotsis@auth.gr

**Keywords:** ISO certification, hospitals, patient safety culture, healthcare professionals, HSOPSC questionnaire

## Abstract

**Background:** ISO certification is widely implemented as a quality assurance tool in healthcare services; however, its impact on patient safety culture (PSC) in public hospitals remains insufficiently explored. **Aim:** This study aims to assess the effect of ISO certification procedures on different dimensions of PSC in public hospital departments by comparing ISO-certified and non-certified departments across two phases (Phase A: pre-certification; Phase B: 18 months post-certification). **Methods:** A two-phase cross-sectional study was conducted in a tertiary public hospital in Greece. Healthcare professionals from both ISO-certified and non-certified departments participated. The Hospital Survey on Patient Safety Culture (HSOPSC v1.0) was administered at two time points (Phase A: baseline, pre-certification; Phase B: 18 months post-certification). A repeated measures analysis was performed to assess the changes over time and differences between the two groups. **Results:** The findings suggest that ISO certification has a mixed impact on the PSC dimensions. A significant improvement was observed in “Supervisor’s/Manager’s Expectations and Actions Promoting Safety” (*p* = 0.012), while “Teamwork Within Units” (*p* = 0.026) and “Handoffs and Transitions” (*p* = 0.037) showed statistically significant changes. These results indicate that certification may enhance structured managerial oversight and interdepartmental collaboration, but at the same time, may negatively impact the teamwork within hospital units. However, no statistically significant changes were observed in “Overall Perception of Safety” (*p* = 0.135) and “Non-Punitive Response to Error” (*p* = 0.101), suggesting that while there was a trend towards a stricter safety evaluation, this was not statistically confirmed. Additionally, the staffing perceptions remained unchanged (*p* = 0.745). **Conclusions:** ISO certification appears to reinforce managerial safety expectations and interdepartmental teamwork, yet does not significantly improve the overall perceptions of patient safety or non-punitive error responses. The results indicate the need for targeted interventions to ensure that certification processes do not increase administrative burdens or negatively impact staff perceptions. Future research should explore whether these effects persist over time and how hospitals can optimize certification processes to strengthen PSC without unintended consequences.

## 1. Introduction

### 1.1. Background and Importance of Patient Safety Culture (PSC)

Patient safety is a fundamental component of high-quality healthcare and is recognized as a global priority by the World Health Organization (WHO) [1]. Strengthening the PSC within hospitals is crucial for improving the healthcare quality and reducing adverse events. PSC encompasses the values, beliefs, and attitudes regarding safety within an organization, influencing how healthcare professionals perceive, respond to, and manage patient safety challenges. A strong PSC has been linked to better patient outcomes, reduced medical errors, and improved teamwork in hospital settings [2,3].

Hospitals with a well-developed PSC are characterized by effective communication, mutual trust, and a proactive approach to risk identification and mitigation [4]. However, despite the ongoing efforts to promote patient safety, adverse events remain a critical issue in healthcare systems worldwide. Recent studies indicate that a positive safety culture is strongly associated with improved adherence to clinical guidelines, higher patient satisfaction, and better staff engagement [5,6].

### 1.2. ISO Certification and Its Role in Healthcare

To enhance the quality and safety, healthcare institutions have adopted various frameworks, including ISO certification, which serves as an internationally recognized quality management system (QMS). ISO 9001, the most widely used QMS standard, focuses on establishing structured policies for risk management, continuous improvement, and patient-centered care [7]. In the healthcare sector, ISO EN 15224:2017 [8], an adaptation of ISO 9001:2015 [9], includes specific requirements for patient safety and clinical risk management [10].

The rationale behind ISO certification in healthcare is that standardized procedures and systematic quality control can lead to better safety outcomes, enhanced operational efficiency, and a reduction in adverse events [11]. However, empirical evidence on its actual impact on PSC remains inconsistent. Some studies report that ISO-certified hospitals demonstrate improved organizational learning, teamwork, and structured safety management [12,13]. In contrast, other studies argue that ISO certification alone does not necessarily lead to improvements in patient safety outcomes and may even increase the administrative burden, leading to unintended consequences for healthcare staff [14]. Furthermore, the effect of ISO certification on various PSC dimensions—such as non-punitive response to error, teamwork, and managerial support—has not been thoroughly examined in public hospital settings [15].

### 1.3. The Need for Evaluating the Impact of ISO Certification on PSC

Considering these conflicting findings, further research is needed to determine whether ISO certification enhances patient safety culture or introduces challenges. This study aims to fill this research gap by assessing the impact of ISO certification across different PSC dimensions in public hospital departments. Specifically, we compare ISO-certified and non-certified departments at two time points—before and after certification—to analyze the changes in key PSC dimensions, including teamwork, managerial expectations, staffing, and non-punitive responses to error.

By systematically examining the two-phase impact of ISO certification, this study provides valuable insights into whether certification effectively strengthens PSC or whether additional interventions are required to mitigate negative consequences and optimize the benefits of certification.

## 2. Materials and Methods

### 2.1. Study Design

A two-phase cross-sectional study was conducted to evaluate the impact of ISO certification on PSC in a tertiary public hospital in Greece. Data collection took place at two time points: Phase A (pre-certification) and Phase B (18 months post-certification). The study included healthcare professionals (HCPs) from both ISO-certified and non-certified departments, enabling a comparative analysis of PSC changes over time.

### 2.2. Sample Size and Participants

Sample size estimation was performed using Stata 12.0, based on prior studies on PSC assessment in healthcare. Assuming a significance level (α = 0.05), a statistical power of 0.80, and an effect size of 0.01, the required sample size per subgroup was N = 219 healthcare professionals.

In Phase A, 441 participants were included (response rate: 68.6%), while in Phase B, 437 professionals completed the follow-up survey (response rate: 63.2%). The sample comprised nurses, physicians, and administrative personnel from both medical and surgical departments, recruited through convenience sampling to ensure adequate representation across clinical units.

### 2.3. Data Collection

Data were collected using the Hospital Survey on Patient Safety Culture (HSOPSC v1.0), a validated instrument widely utilized in healthcare safety research. The survey was distributed in sealed envelopes and returned to secure collection boxes to maintain anonymity. No personal identifiers were recorded. A cover letter outlined the study objectives and voluntary participation.

PSC perceptions were assessed as follows:

Phase A (Baseline): Among both departments undergoing ISO certification and non-certified departments.

Phase B (18 months post-certification): A follow-up assessment to examine the changes in PSC dimensions.

### 2.4. Measurement Tool (HSOPSC v1.0)

The Greek version of HSOPSC v1.0 was used. The questionnaire consists of 42 items across 12 PSC dimensions, including “Communication Openness”, “Feedback and Communication About Errors”, “Staffing”, “Teamwork Within Units”, “Teamwork Across Units”, and “Overall Perception of Safety”.

The responses were recorded on a 5-point Likert scale (1 = strongly disagree, 5 = strongly agree), with higher scores reflecting stronger perceptions of patient safety culture.

### 2.5. Statistical Analysis

Data were analyzed using SPSS v26.0 (IBM, Armonk, NY, USA).

Descriptive statistics were computed for categorical variables (frequencies, percentages) and continuous variables (means, standard deviations, minimum, and maximum values).

Repeated measures analysis of variance (RM-ANOVA) was conducted to assess the changes in PSC dimensions over time (Phase A vs. Phase B) and between groups (ISO-certified vs. non-certified departments).

Pairwise comparisons were adjusted using the Bonferroni correction.

Partial Eta Squared (η^2^) was calculated to determine effect sizes:

η^2^ < 0.01 = negligible effect

η^2^ 0.01–0.06 = small effect

η^2^ 0.06–0.14 = moderate effect

η^2^ > 0.14 = large effect

Statistical significance was set at *p* < 0.05.

Data visualization and statistical comparisons were performed using Python Matplotlib 3.10.1 and IBM SPSS v26.0 for statistical verification.

## 3. Results

### 3.1. Description of the Participants

#### 3.1.1. Phase A

A total of 441 health workers (nursing, medical, and administrative staff) participated in the study (response rate 68.6%), with a mean age of 43.3 years (SD: 9.4, min: 19, max: 65). The participants had been employed in the specific hospital for a mean of 10.99 years (SD: 7.66, min: 1, max: 25), and 6.77 years (SD: 6.46, min: 1, max: 23) in the specific department. They worked a mean of 42.65 h per week (SD: 9.17, min: 20, max: 112), while their total professional experience was 14.05 years (SD: 9.57, min: 1, max: 40).

#### 3.1.2. Phase B

A total of 437 health workers (nursing, medical, and administrative staff) participated in the study (response rate 63.2%), with a mean age of 44.04 years (SD: 9.16, min: 20, max: 65). The participants had been employed in the specific hospital for a mean of 12.22 years (SD: 8.09, min: 1, max: 25) and 7.99 years (SD: 6.82, min: 1, max: 25) in the specific department. They worked a mean of 43.38 h per week (SD: 8.73, min: 6, max: 100), while their total professional experience was 14.79 years (SD: 9.27, min: 1, max: 38).

The descriptive statistics of the study sample in Phase A and Phase B are presented in detail in Table 1.

### 3.2. Results from the Two-Phase Comparison Between Subgroups

The comparison of PSC dimensions between ISO-certified and non-certified departments across the two phases revealed a mixed impact of ISO certification. Statistically significant improvements were observed in “Supervisor’s/Manager’s Expectations and Actions Promoting Patient Safety” (*p* = 0.012) and “Handoffs and Transitions” (*p* = 0.037), suggesting that certification enhanced managerial oversight and interdepartmental communication. “Teamwork Within Units” also showed a significant variation (*p* = 0.026), indicating potential shifts in intra-departmental dynamics following certification.

However, “Overall Perception of Safety” (*p* = 0.135) and “Non-Punitive Response to Error” (*p* = 0.101) did not demonstrate statistically significant changes, implying that while procedural modifications occurred, staff perceptions of safety culture remained largely unchanged. Additionally, staffing perceptions (*p* = 0.745) showed no improvement, highlighting persistent concerns regarding workforce sufficiency despite certification efforts.

These findings suggest that ISO certification may strengthen structured safety management and interdepartmental collaboration but does not necessarily translate into enhanced frontline safety perceptions (Table 2). Further institutional measures, such as targeted staff training and leadership engagement, may be required to maximize the benefits of certification while addressing workforce challenges.

**Figure 1:**
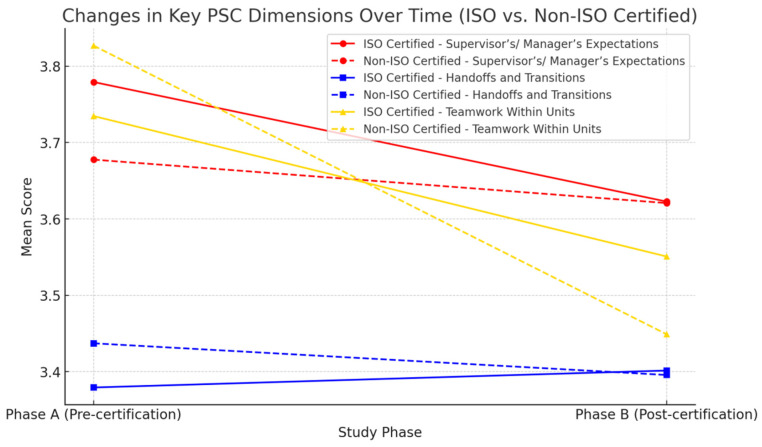
Impact of ISO certification on key dimensions of patient safety culture: supervisor expectations, handoffs, and teamwork.

Figure 1 illustrates the changes in the three key dimensions of patient safety culture (PSC) as influenced by ISO certification: “Supervisor’s/Manager’s Expectations and Actions Promoting Safety”, “Handoffs and Transitions”, and “Teamwork Within Units”. The analysis compares two groups, ISO-certified and non-certified hospital departments, across two time points: Phase A, prior to certification, and Phase B, 18 months after certification.

The results suggest that ISO certification enhances structured leadership in patient safety management. A notable increase is observed in “Supervisor’s/Manager’s Expectations and Actions Promoting Safety” among ISO-certified departments, which remains stable in Phase B. This trend indicates that certification contributes to a more systematic and structured approach to patient safety through managerial oversight and clearly defined safety expectations. Similarly, “Handoffs and Transitions” show improvement in the ISO-certified group, suggesting that standardization through certification enhances the communication between hospital units, a finding aligned with prior research on quality management systems in healthcare.

In contrast, the dimension of “Teamwork Within Units” presents a more complex pattern. While the initial scores were comparable between the two groups, in Phase B, the teamwork perceptions in ISO-certified departments remained stable, whereas a slight decline was noted in non-certified departments. This finding implies that while ISO certification may strengthen interdepartmental communication and structured workflows, its effect on intra-unit teamwork is less pronounced. Previous studies have indicated that the teamwork dynamics within hospital units are influenced by multiple factors beyond procedural standardization, including leadership styles, workload distribution, and organizational culture.

These findings support the hypothesis that ISO certification reinforces structured managerial approaches and improves the efficiency of interdepartmental patient handoffs but does not necessarily enhance the teamwork within individual hospital units. This aligns with prior literature suggesting that while ISO-driven quality management systems promote adherence to standardized protocols, additional interventions, such as leadership development programs and team-based training, may be required to improve the internal team dynamics.

The graphical representation of these results provides a clear and accessible means for understanding the impact of ISO certification on key PSC dimensions. The observed trends highlight both the benefits and limitations of certification in shaping safety culture, reinforcing the need for complementary strategies to optimize the teamwork within hospital units while sustaining the positive effects of structured leadership and improved handoff processes.

## 4. Discussion

This study assessed the impact of ISO certification on PSC in a tertiary public hospital, revealing both improvements and challenges. The findings suggest that while certification enhances structured managerial oversight and interdepartmental teamwork, it does not necessarily lead to improved frontline staff perceptions of patient safety. These results align with previous research examining the effectiveness of quality management systems and accreditation programs in healthcare settings, which indicate that certification contributes to process standardization but may have a limited influence on the subjective perceptions of safety among healthcare professionals [16,17].

A significant improvement was observed in “Supervisor’s/Manager’s Expectations and Actions Promoting Patient Safety” (*p* = 0.012) and “Handoffs and Transitions” (*p* = 0.037). These findings suggest that ISO certification reinforces the structured leadership in patient safety management while also improving the communication between hospital units. Similar trends have been reported in studies evaluating the impact of hospital accreditation and ISO certification, where structured quality frameworks have been linked to improved managerial accountability and team-based coordination [13,18]. The improvement in teamwork across units further supports the notion that ISO certification fosters interdepartmental collaboration by promoting standardized communication protocols and safety compliance [19,20]. Studies conducted in European countries, Australia, and Saudi Arabia confirm that hospitals implementing ISO-based quality management systems demonstrate enhanced cross-departmental teamwork and adherence to procedural guidelines, leading to a more cohesive approach to patient safety [21,22].

Despite these positive outcomes, the study also identified areas where ISO certification did not lead to significant improvements or resulted in declining perceptions of certain PSC dimensions. No statistically significant change was observed in “Overall Perception of Safety” (*p* = 0.135), suggesting that certification alone does not inherently enhance staff confidence in safety conditions. Similar findings have been reported in studies, where hospital accreditation improved the structural safety processes but had minimal effects on how staff perceived the overall safety culture [15,23]. Additionally, ”Non-Punitive Response to Error” remained statistically unchanged (*p* = 0.101), indicating that ISO certification did not adequately address concerns related to the fear of blame for errors. The persistence of this issue is consistent with findings from other studies, which suggest that organizations undergoing rigorous quality assessments often experience an initial decline in error reporting due to the increased scrutiny and accountability pressures [24,25].

Another key finding was the absence of improvement in staffing perceptions (*p* = 0.745), reinforcing concerns that ISO certification does not address fundamental workforce sufficiency issues. This is a significant challenge, as previous research has shown that accreditation and certification processes frequently introduce additional administrative burdens without resolving the existing staffing shortages [26]. Studies on hospital accreditation programs in Jordan and Denmark have similarly demonstrated that while certification improves process efficiency, it does not alleviate the workload pressures faced by healthcare professionals, which remain a primary barrier to sustained patient safety improvements [27,28].

The findings of this study contribute to the ongoing debate regarding the effectiveness of ISO certification in healthcare. While structured quality management systems enhance regulatory compliance and managerial oversight, they do not necessarily lead to an improved perception of safety culture among healthcare workers. One possible explanation is that ISO certification introduces stricter evaluation criteria, leading staff to assess hospital safety conditions more critically. This phenomenon has been observed in hospitals undergoing accreditation-based quality audits, where structured risk assessments resulted in more rigorous internal evaluations of patient safety performance [23,29]. Furthermore, the lack of improvement in staffing perceptions suggests that ISO certification alone is insufficient to address workforce-related challenges and resource allocation concerns. Similar findings have been reported in accreditation studies across European and Middle Eastern hospitals, where persistent staffing shortages have remained a critical issue despite the implementation of standardized safety protocols [30,31].

Future studies could explore whether the observed effects persist over a longer period and how specific leadership interventions might address intra-unit teamwork challenges. Additionally, investigating the role of staff engagement and training programs in optimizing the benefits of ISO certification would provide further insights into sustaining a positive patient safety culture over time.

To maximize the benefits of ISO certification while addressing its limitations, hospital administrators should consider implementing complementary interventions. Efforts should focus on minimizing the administrative burdens associated with certification to ensure that compliance processes do not divert attention from direct patient care responsibilities. Additionally, fostering a non-punitive culture of safety is essential, as ensuring that incident reporting occurs without the fear of disciplinary action is a key determinant of PSC sustainability. Strengthening intra-unit teamwork and staff engagement initiatives may further mitigate the observed limitations in teamwork and safety perceptions. Future research should explore whether the observed effects persist over time and how hospitals can optimize the certification processes to enhance PSC without introducing operational inefficiencies. Comparative analyses between ISO-certified and accredited hospitals could provide valuable insights into which quality improvement strategies are most effective in fostering a sustainable patient safety culture.

## 5. Conclusions

This study evaluated the impact of ISO certification on PSC in a tertiary public hospital, revealing both improvements and persistent challenges. The findings indicate that ISO certification contributes to structured managerial oversight and interdepartmental collaboration, particularly in enhancing supervisor engagement in patient safety and improving handoffs and transitions. These results are consistent with previous research suggesting that standardized quality management systems facilitate procedural adherence and foster a culture of safety within healthcare institutions.

However, the study also highlights critical limitations. No statistically significant changes were observed in the overall perceptions of safety or non-punitive responses to error, suggesting that ISO certification alone may not be sufficient to alter frontline staff attitudes toward patient safety. Furthermore, staffing perceptions remained unchanged, reinforcing the concerns that certification processes do not inherently address workforce shortages or administrative burdens. These findings align with prior research indicating that while ISO certification provides a structured framework for quality improvement, its effectiveness depends on comprehensive institutional engagement and ongoing workforce support.

Given these insights, hospital administrators should consider integrating ISO certification with complementary interventions aimed at reinforcing patient safety culture. Strategies such as leadership-driven safety initiatives, enhanced staff training programs, and policies promoting non-punitive error reporting may help maximize the impact of the certification efforts. Future research should focus on longitudinal assessments of ISO-certified hospitals to determine whether observed effects persist over time and to identify the best practices for optimizing the certification outcomes. Comparative analyses between ISO-certified and accredited hospitals could further clarify the relative advantages and limitations of different quality assurance frameworks in healthcare settings.

These findings reinforce the notion that while ISO certification can serve as a valuable structural framework for enhancing managerial oversight and interdepartmental collaboration, it should be complemented by targeted interventions that directly support frontline staff. A multi-faceted approach, integrating leadership training, continuous staff engagement, and a non-punitive culture of safety, may be required to fully realize the benefits of certification in hospital settings.

While ISO certification provides a structured approach to quality and safety management, its full potential can only be realized through a broader institutional commitment to continuous improvement, staff engagement, and patient-centered care.

## Figures and Tables

**Table 1:** Demographic and occupational data for the total samples in both phases of the study.

	Phases							
	Phase A				Phase B			
	N		%		N		%	
Gender								
Male	78		17.7%		94		21.5%	
Female	363		82.3%		343		78.5%	
Total	441		100%		437		100%	
Participants in work environment based on ISO certification process								
Participants of departments in ISO certification process	230		52.2%		222		50.8%	
Participants of Non-ISO certified departments	211		47.8%		215		49.2%	
Total	441		100%		437		100%	
	**Min.**	**Max.**	**Mean**	**Standard Deviation (SD)**	**Min.**	**Max.**	**Mean**	**Standard Deviation (SD)**
Age	19	65	43.34	9.40	20	65	44.04	9.16
How many years have you worked in this hospital?	1	25	10.99	7.66	1	50	12.22	8.09
How many years have you worked in this specific department?	1	23	6.77	6.46	1	25	7.99	6.82
Working hours per week	20	112	42.65	9.17	6	100	43.38	8.73
Total time of professional experience	1	40	14.05	9.57	1	38	14.79	9.27

**Table 2:** Comparison of patient safety perceptions between ISO-certified and non-ISO-certified departments across two phases.

	Phases				*p*-Value (Phase * Groupsv2)	F-Value	Partial Eta Squared
	Phase A		Phase B				
	Non-ISO Certification Departments	ISO-Certified Departments	Non-ISO Certification Departments	ISO-Certified Departments			
	Mean	Mean	Mean	Mean			
Communication Openness	3.29	3.38	3.27	3.24	0.25	1.328	0.002
Non-Punitive Response to Error	2.79	2.83	2.66	2.52	0.101	2.69	0.003
Feedback and Communication About Error	3.40	3.38	3.36	3.39	0.135	2.233	0.003
Staffing	2.49	2.55	2.44	2.38	0.745	0.106	<0.001
Management Support for Patient Safety	3.44	3.41	3.32	3.32	0.28	1.166	0.001
Supervisor’s/Manager’s Expectations and Actions Promoting Safety	3.68	3.78	3.62	3.62	0.012 *	6.411	0.007
Organizational Learning—Continuous Improvement	3.70	3.56	3.61	3.66	0.876	0.024	<0.001
Feedback and Communication About Error	3.54	3.64	3.55	3.66	0.456	0.557	0.001
Handoffs and Transitions	3.44	3.38	3.40	3.40	0.037 *	4.378	0.005
Teamwork Within Units	3.83	3.73	3.45	3.55	0.026 *	5.005	0.006
Teamwork Across Units	3.40	3.33	3.35	3.46	0.132	2.27	0.003
Overall Perception of Safety	3.63	3.61	3.46	3.57	0.135	2.233	0.003

* *p* < 0.05.

## Data Availability

Data are provided within the manuscript.
